# Low Intensity Exercise Training Improves Skeletal Muscle Regeneration Potential

**DOI:** 10.3389/fphys.2015.00399

**Published:** 2015-12-24

**Authors:** Tiziana Pietrangelo, Ester S. Di Filippo, Rosa Mancinelli, Christian Doria, Alessio Rotini, Giorgio Fanò-Illic, Stefania Fulle

**Affiliations:** ^1^Department of Neuroscience, Imaging and Clinical Sciences, University “G. d'Annunzio” Chieti-PescaraChieti, Italy; ^2^Laboratory of Functional Evaluation, “G. d'Annunzio” University of Chieti-PescaraChieti, Italy; ^3^Centre for Aging Sciences, d'Annunzio FoundationChieti, Italy; ^4^Department of Neuroscience, Imaging and Clinical Sciences, Interuniversity Institute of MyologyChieti, Italy

**Keywords:** low-to-moderate intensity exercise training, satellite cells, superoxide anion, oxidative status, miRNA, women

## Abstract

**Purpose:** The aim of this study was to determine whether 12 days of low-to-moderate exercise training at low altitude (598 m a.s.l.) improves skeletal muscle regeneration in sedentary adult women.

**Methods:** Satellite cells were obtained from the *vastus lateralis* skeletal muscle of seven women before and after this exercise training at low altitude. They were investigated for differentiation aspects, superoxide anion production, antioxidant enzymes, mitochondrial potential variation after a depolarizing insult, intracellular Ca^2+^ concentrations, and micro (mi)RNA expression (miR-1, miR-133, miR-206).

**Results:** In these myogenic populations of adult stem cells, those obtained after exercise training, showed increased Fusion Index and intracellular Ca^2+^ concentrations. This exercise training also generally reduced superoxide anion production in cells (by 12–67%), although not in two women, where there was an increase of ~15% along with a reduced superoxide dismutase activity. miRNA expression showed an exercise-induced epigenetic transcription profile that was specific according to the reduced or increased superoxide anion production of the cells.

**Conclusions:** The present study shows that low-to-moderate exercise training at low altitude improves the regenerative capacity of skeletal muscle in adult women. The differentiation of cells was favored by increased intracellular calcium concentration and increased the fusion index. This low-to-moderate training at low altitude also depicted the epigenetic signature of cells.

## Introduction

Satellite cells are myogenic cells that are responsible for postnatal skeletal muscle growth. In normal adult muscle, satellite cells account for differently activated peripheral sub-sarcolemmal nuclei, which depend on the metabolic properties of the muscle fiber and the age of the person (Verdijk et al., 2014). In response to various stimuli, satellite cells can enter the mitotic cycle, proliferate, and fuse, thereby contributing to muscle regeneration for repair or hypertrophy of postnatal skeletal muscle (Lorenzon et al., 2004; Snijders et al., 2012; Ceafalan et al., 2014). Satellite cells are specifically involved in skeletal muscle adaptation to different types of exercise, such as with strength (Kvorning et al., 2014; Verdijk et al., 2014) and endurance (Kadi et al., 2004) training, whereby the intensity and duration of muscle stimulation is crucial for satellite cell activation. Indeed, although it has been demonstrated that satellite cells are not activated in response to a single bout of exercise (Kadi et al., 2004), they can modulate specific factor content after 9 h of combined resistance–endurance exercise (Verdijk, 2014). Furthermore, even if it is currently accepted that exercise has positive effects on skeletal muscle regeneration, the fitness level of subjects and the type and intensity of exercise protocols have crucial roles in satellite cell activation. In fact, a number of study demonstrated that both resistance and endurance training increased satellite cells content (Kadi et al., 2005). At the ultrastructural level, it has been observed that the endurance-training programme induced the formation of new myotubes (Appell et al., 1988). However, there are many points to be addressed at molecular level. The [Ca^2+^]i increase is a prerequisite for fusion process due to specific signaling it activates (Millay et al., 2013; Hindi et al., 2013). In fact, many studies have shown that myoblast fusion is regulated by [Ca^2+^]i increase (Constantin et al., 1996) that may depend on cholinergic (Bernareggi et al., 2012), stretch-activated and KCa channels activation (Pietrangelo et al., 2006; Shin et al., 1996).

Another molecular messenger influenced by exercise is reactive oxygen species (ROS) production (Abruzzo et al., 2013). The ROS include: superoxide anions, hydroxyl radicals, oxide anions, hydrogen peroxide, nitric oxide, peroxynitrite, lipid peroxyls, and lipid alkoxyls. ROS production is related to with the term oxidative stress, which was originally defined as “a disturbance in the pro-oxidant/anti-oxidant balance in favor of the former” (Siens and Cadenas, 1985). However, due to the complexity of the cellular redox balance, this was refined to “an imbalance between oxidants and anti-oxidants in favor of the oxidants, leading to a disruption of redox signaling and control and/or molecular damage” (Siens and Jones, 2007; Powers et al., 2011).

The cellular antioxidant system consists on the activity of scavengers as vitamin C and E, for instance, and enzymes as glutathione peroxidase, superoxide dismutase (SOD), catalase (Cat). In particular, the SOD reduces the superoxide anion to hydrogen peroxide and in turn the Cat reduces this to water. There are several studies accounting for the involvement of antioxidant enzymes in exercise-induced muscle plasticity and also in vitamin supplementation (Cumming et al., 2014; Nikolaidis et al., 2015). However, it is not well understood the role of antioxidants in human myogenesis.

In mitochondria the cellular aerobic metabolism reduces around 1–2% of oxygen to superoxide anions, which represent the most abundant free radicals produced. The superoxide reactivity could last for days in absence of enzymatic removal and it can spread out into also outside the cell, and undergo reactions far from its site of production, thus provoking cellular and general oxidative stress. However, a gender distinguish has to be considered, as female subjects are more protected than men against oxidative stress thanks to their estrogen hormone. The estrogen level in young woman exerts an antioxidant effect, as demonstrated by four-fold less DNA and lipid oxidation in female with respect to male subjects (Mecocci et al., 1999; Green and Simpkins, 2000).

Skeletal muscle contraction during exercise produces variable amounts of ROS, which depend on exercise intensity and duration (Fisher-Wellman and Bloomer, 2009; Powers et al., 2011). ROS can activate specific signaling pathways at the plasma membrane and/or stimulate gene transcription (Gundersen, 2011; Baar, 2014). In particular, recent literature suggests that both exercise and ROS can activate muscle-specific microRNAs (myo-miRs; small post-transcriptional RNAs), and regulate the differentiation levels of satellite cells (Eisenberg et al., 2009; Crippa et al., 2012; Huang et al., 2012). Among the myo-miRs of value for satellite cells, there are miR-1, miR-133, and miR-206 (Kwon et al., 2005; McCarthy and Esser, 2007; La Rovere et al., 2014).

The aim of this study was to determine whether 12-day exercise training at low altitude (598 m a.s.l.) can improve skeletal muscle regeneration in adult women. In particular, we investigated whether this kind of exercise could affect some molecular actor of the differentiation process as the fusion index, the intracellular calcium level, the redox balance, the mitochondrial activation, and the miRNA expression.

## Methods

### Subjects

Seven healthy women of childbearing age (mean age, 36.3 ± 7.1 years old) who were generally used to a sedentary life-style (at 110 m altitude sea level, a.s.l.) were enrolled to serve as subjects to the study known as GOKYO KHUMBU/AMA DABLAM TREK 2012. None of these women suffered from any metabolic or skeletal muscle diseases. The women were not engaged in any specific trekking or exercise training protocols within a few months of their enrolment, except two of them who occasionally went trekking. All of the subjects provided written, informed consent before participating in the study. The study was conducted according to the Helsinki Declaration, and it was approved by Ethic Committee of “G. d'Annunzio” University of Chieti-Pescara, Italy (protocol no. 773 COET).

### Experimental design and training

Trekking consisted of a 12- day walking at low-altitude on mountain paths in central Italy (L'Aquila, Abruzzo, Italy). The average altitude was 598 m ± 561 and a range of difference in elevation between consecutive days was 250–1000 m. The total covered distance was 139600 m. The total ascent and descent were 5500 m (458 m d^−1^; range: 0–1000 m d^−1^) and 5350 m (445 m d^−1^; range: 0–1000 m d^−1^), respectively. The total walking time was 149580 s ± 27.06, on average 3 h and 28 min per day, and the average speed was 0.93 m s^−1^. The total number of steps was 182372 ± 77.43. The volunteers freely choose their intensity of exercise also considering the general recommendations to approach physical exercise with a load adapted to personal capacity (guidelines of American College Sport Medicine, Garber et al., 2011). The exercise intensity of the training was monitored with a heart rate monitor for each subject (POLAR®, Kempele, Finland). The average heart rate of the seven subjects in the 12 day of exercise training period was 111 ± 10 bpm, which was classified as light-to-moderate intensity (Tam et al., 2015). The subjects did not perform other exercise outside the trekking protocol.

### Skeletal muscle needle biopsy

Tiny percutaneous needle biopsies from the *vastus lateralis* muscle were performed at the Laboratory of Functional Evaluation, “G. d'Annunzio” University of Chieti-Pescara, as described by Pietrangelo et al. (2011), a week before initiating the exercise training (PRE-Ex), and 9 days after the specific planned light-to-moderate exercise training at low altitude (POST-Ex). Specifically, after the training period, the subjects stayed at rest for a couple of days, then they were engaged in functional evaluations described in (Tam et al., 2015), that lasted 5 days, and after a couple of days of recovering, they had the needle biopsies.

### Satellite cell population and myogenicity

The satellite cells were obtained, expanded as myoblasts in growth medium, and differentiated as previously described (Fulle et al., 2005; Mancinelli et al., 2011). Briefly, the percentages of myogenicity of the cell cultures were obtained using an immunocytochemistry assay, with the marker desmin (Kaufman and Foster, 1988; Behr et al., 1994), and with biotinylated streptavidin-AP kits (LSAB + System-AP Universal kits; Cat. No. K0678; DAKO, Dakocytomation, Glostrup, Denmark). Differentiation of the cell populations was determined by counting the numbers of nuclei in the myotubes after 7 days of differentiation, as percentages with respect to the total number of nuclei, with the ratio between these two values (nuclei in myotubes/total nuclei × 100%) giving the Fusion Index. We only considered myotubes that were positive to the primary antibody against myosin heavy chain (MHC), using the MF20 anti-MHC monoclonal antibody (diluted 1:50; Developmental Studies Hybridoma Bank, University of Iowa, Iowa City, IA, USA), and that contained three or more nuclei (Pietrangelo et al., 2009).

### Intracellular calcium concentration measurement

The cells were loaded with Fura2-AM at the final concentration of 5 μM for 30 min, which was then de-esterificated for 20 min at 37°C. The experiments were performed and images were acquired using the procedures and set-up described by Pietrangelo et al. (2002).

### Reactive oxygen species

The general analysis of ROS, specifically the cellular peroxidation end products, was conducted using the dye 2,7-dichlorofluorescein diacetate (DCF; Cat.No. D6883; Sigma). The cells were plated and grown in 96-well microplates (1000 cells 0.32 cm^−1^), and incubated with 10 μM DCF for 30 min at 37°C in sterile normal extracellular solution (140 mM NaCl, 2.8 mM KCl, 2 mM CaCl_2_, 2 mM MgCl_2_, 10 mM glucose, 10 mM Hepes, pH 7.3). The fluorescence of the dye accumulated in the cytoplasm (i.e., 2,7-dichlorofluorescein) was determined at 530 nm (excitation, 490 nm) using a fluorometer (SPECTRAmax Gemini XS; Molecular Devices Toronto, ON, Canada). The analysis was conducted using the SOFTmax Pro software. The cells were stimulated with 100 nM hydrogen peroxide (H_2_O_2_) to evaluate their response to an oxidant (Menghini et al., 2011).

To determine the superoxide anion (${\text{O}}_{2}^{•-}$), we used an assay based on the dye nitroblue tetrazolium chloride (NBT; Cat. No. N6639; Sigma-Aldrich) and its reduction into formazan ${\text{O}}_{2}^{•-}$ (Sozio et al., 2013). The absorbance at 550 nm was determined using a spectrophotometer (SPECTRAmax 190 microplate 257; Molecular Devices, Sunnyvale, CA, USA), such that the greater the ${\text{O}}_{2}^{•-}$ level, the greater the absorbance. The cells (1 × 10^6^ cells) were detached, centrifuged at 170 × *g* for 5 min, resuspended in 1 ml NBT at 1 mg ml^−1^ in 0.9% aqueous NaCl, and incubated for 3 h at 37°C. Then, the cells were centrifuged at 100 × *g* for 10 min, resuspended in 1 ml DMSO, and left for 20 min at 37°C. Finally, the NBT absorbance was determined.

### Transmembrane mitochondrial potential

The mitochondrial membrane potential was determined using the JC-1 dye (5,5′,6, 6′-tetracloro-1,1′,3,3′-tetraethylbenzimidazolylcarbocianine iodide/chloride; Molecular Probes). JC-1 is a cationic dye that accumulates in the mitochondria. When the mitochondrial potential is high, as in normal cells, JC-1 aggregates into dimers that emit red fluorescence (aggregated J: excitation/ emission, 560/595 nm). When the membrane potential is low, as in the presence of oxidative stress, JC-1 forms monomers that emit green fluorescence (excitation/emission, 488/522 nm), with concomitant decreased red fluorescence. The ratio of the red/green fluorescence depends exclusively on the mitochondrial potential, with no effects of other factors (such as mitochondrial dimension, volume, shape, or density). The cells were plated into 96-well plates, incubated with 10 μg ml^−1^ JC-1 for 15 min at 37°C, and assayed using a fluorometer (SPECTRAmax Gemini XS; Molecular Devices Toronto, ON, Canada) equipped with the SoftMax Pro software (Gemini XS, Molecular Devices Toronto, ON, Canada) (Nuydens et al., 1999). The fluorescence is reported as means ±SEM of the red/green fluorescence ratios of samples with respect to control, as f(r/g)/f(r/g)_c_ (Morabito et al., 2010), and is here given as ΔΨ_mit_. The dye ratio for JC-1 between the inner and outer mitochondrial membrane potentials was related to the mitochondrial depolarization after an oxidant insult, such as with (H_2_O_2_).

### Antioxidant enzyme activity

The antioxidant enzymes analyzed were superoxide dismutase and catalase, The assays were performed using the cells cytosolic fraction.

### Superoxide dismutase

The activity of superoxide dismutase (SOD) is direct against ${\text{O}}_{2}^{•-}$. SOD catalyzes a disproportionation reaction where a first ${\text{O}}_{2}^{•-}$ is oxidized and the second molecule is reduced, turning two molecules of superoxide into O_2_ and H_2_O_2_.The enzymatic activity was determined according to Fulle et al. (2000) The final assay volume was 1 ml and contained 20 mM Na_2_CO_3_ buffer pH 10, 10 mM Cytochrome c, 1 mM Xanthine and Xanthine Oxidase. Xanthine-xanthine oxidase is the ${\text{O}}_{2}^{•-}$ generation system. As the xanthine oxidase activity varies, the amount used for the assay was such that produced a rate of cytochrome c reduction, at 550 nm, of 0.025 per minute without SOD addiction. The assay was performed at 550 nm for 10 min. The SOD units were calculated considering that 1 SOD unit is defined as the quantity that inhibits the rate of cytochrome c reduction by 50%.

### Catalase

The reaction for which catalase (Cat) is best known is the “catalatic” reaction, in which H_2_O_2_ oxidizes the heme iron of the resting enzyme to form an oxyferryl group with a π-cationic porphyrin radical (Kirkman and Gaetani, 2006). This step is followed by oxidation of a second molecule of H_2_O_2_. Catalase forms two molecules of H_2_O and O_2_, starting from two molecules of H_2_O_2_. Catalase activity was determined, according to Greenwald (1985), by the decrease in absorbance due to H_2_O_2_ consumption (ε = −0.04 mM^−1^ cm^−1^) measured at 240 nm. The final reaction volume was 1 ml and contained 100 mM Na-phosphate buffer pH 7.0, 12 μM H_2_O_2_ and 70 μg of sample proteins. The reaction was followed for 1 min and the Cat activity was expressed in μmol/minute/mg proteins.

### miRNA expression

PureLink miRNA Isolation kits were used for the miRNA extractions (Cat. No. K1570-01, Invitrogen, Life Technologies, Molecular Devices, Sunnyvale, USA). About 800,000 cells were resuspended in 300 μl binding buffer (from the PureLink miRNA kits), and 300 μl 70% alcohol was added to the lysate. This was forced into the spin cartridges of the PureLink miRNA Isolation kits, which were then centrifuged at 12000 × *g* for 1 min. After washing with 100% alcohol, these were centrifuged again, as before. Then 500 μl wash buffer was added to the spin cartridges, which were centrifuged again at 12000 × *g* for 1 min. This procedure was performed twice, and then the spin cartridges were centrifuged at 12000 × *g* for 3 min, to remove residual buffer. Finally, they were eluted with 50 μl RNase-free sterile water. The RNA concentrations were determined using a NanoDrop™ spectrophotometer.

Retro-transcription and real-time PCR were carried out according to the Applied Biosystems TaqMan miRNA assay kit protocols. Briefly, the retro-transcription involved 20 ng of a “small” RNA, as the “stem loop” primer that was specific for each miRNA, dNTPs, and inverse transcriptase RNAse inhibitors (according to the Applied Biosystems high capacity cDNA reverse transcription kit, part N° 4368814), using a thermocycler (30 min at 16°C, 30 min at 42°C, 5 min at 85°C, then at 4°C). Then, the real-time PCR for the miRNA expression levels was performed using TaqMan probes and specific TaqMan®Universal Master Mix II, without UNG, in 96-well plates (Part No.: 4440040, Applied Biosystems) with a sequence detection system (Applied Biosystems PRISM 7900 HT), in triplicate. MiR-16 was used as the endogenous control. The specific miRNA sequence probes used were (Applied Biosystems):

- has-miR-1 (UGGAAUGUAAAGAAGUAUGUAU; #002222);
- has-miR-206 (UGGAAUGUAAGGAAGUGUGUGG; #000510);
- has-miR-133b (UUUGGUCCCCUUCAACCAGCUA; #002247);
- has-miR-16-5p (UAGCAGCACGUAAAUAUUGGCG; #000391).

The relative quantification of the miRNA targets was carried out using the ΔCt formula, according to the Ct method.

### Statistical analysis

The statistical analysis was carried out using GraphPad Prism Software, version 5 (GraphPad Software, La Jolla, CA, USA). The data are reported as means ± standard error (SE). Unpaired and paired *t*-tests (for different group of cells and for the same cells with specific treatment, respectively) were used to reveal the statistical differences.

## Results

### Subjects

Seven healthy women of childbearing age who were generally used to a sedentary life-style were enrolled to study the skeletal muscle regeneration potential after low to moderate intensity training as trekking at low altitude. Table 1 summarize their anthopometric and physiological features.

**Table 1:** **Anthropometric and physiological characteristics of the subjects before (PRE-Exercise) and after (POST-Exercise) 12-days training period**.

	**PRE-exercise**	**POST-exercise**
BW (Kg)	65.7 ± 4.4	65.1 ± 4.0
BMI (Kg m^−2^)	24.3 ± 1.5	24.1 ± 1.4
BF (%)	27.2 ± 2.6	25.9 ± 2.7
VO_2*max*_(L min^−1^)	2.13 ± 0.13	2.16 ± 0.12

*BW, body weight; BMI, body mass index; BF (%), body fat percentage; VO_2max_, maximum oxygen consumption*.

#### Myogenic characteristics and analysis of cell differentiation after exercise training

We obtained myogenic populations of adult stem cells, myoblasts, from percutaneous needle biopsies from the *vastus lateralis* muscle of female volunteers before (PRE-Ex) and after (POST-Ex) low altitude exercise training in the Abruzzo mountains. The characteristics of the cell differentiation are reported in Table 2.

**Table 2:** **Characteristics of myogenicity and differentiation of the satellite cell populations isolated from the seven women after trekking in the Abruzzo hills (central Italy)**.

**Subject**	**PRE-exercise**			**POST-exercise**		
**code**	**%Desmin^+^**	**Fusion Index (%)**	**%Desmin^+^ unfused**	**%Desmin^+^**	**Fusion Index (%)**	**%Desmin^+^ unfused**
#1	68.5	38.0	75.0	70.5	33.3	75.8
#2	66.0	23.3	73.9	77.3	60.2	22.4
#3	76.2	28.2	91.3	87.0	30.0	87.0
#4	90.4	14.6	82.1	88.8	67.9	28.5
#5	70.0	19.8	71.8	46.7	44.5	37.2
#6	67.0	23.5	58.4	34.5	42.6	25.7
#7	48.1	18.0	49.2	–	–	–

*POST-Exercise Fusion Index differs significantly from the PRE-Exercise Fusion Index (p < 0.05, one tailed); POST-Exercise %Desmin^+^ unfused differs significantly from the PRE-Exercise %Desmin^+^ unfused (p < 0.05, one tailed)*.

The analysis of desmin-positive undifferentiated cells suggested that there were no significant difference in the myogenicity between the PRE-Ex and POST-Ex conditions. Of note, the Fusion Index, which represents the percentage of myoblasts that can fuse over 7 days of differentiation forming myotubes, was significantly increased at POST-Ex (*p* < 0.05). The percentage of desmin-positive cells in the differentiation media (after 7 days of differentiation) significantly decreased (*p* < 0.05).

#### Intracellular Ca^2+^ concentrations of cells

**Figure 1:**
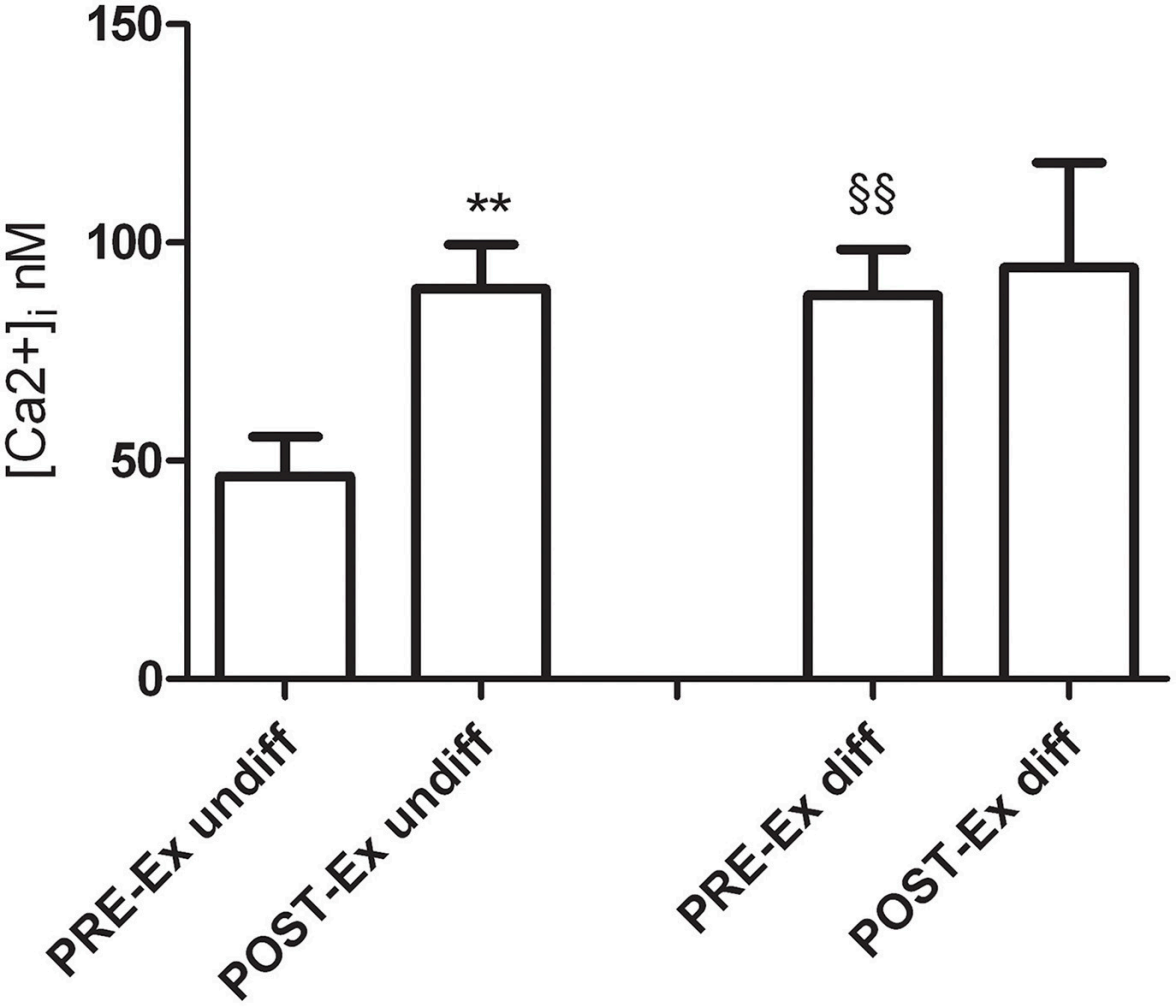
**Intracellular Ca^2+^ concentrations as basal levels for undifferentiated and differentiated (as indicated) cells obtained from skeletal muscle of female subjects for PRE-Ex and POST-Ex (as indicated)**. ^**^ *p* < 0.01 vs. undifferentiated PRE-Ex cells; §§*p* < 0.01 vs. undifferentiated PRE-Ex cells. The total analyzed cells were 90 myoblasts and 70 myotubes.

Figure 1 shows the basal levels of the intracellular Ca^2+^ concentrations ([Ca^2+^]_i_) of the undifferentiated and differentiated cells. The undifferentiated POST-Ex myoblasts had [Ca^2+^]_i_ that were significantly higher than PRE-Ex myoblasts (*p* ≤ 0.01). In the comparison of the differentiated PRE-Ex cells with respect to the undifferentiated PRE-Ex cells, these also showed an increase in [Ca^2+^]_i_ (*p* ≤ 0.01). There were, however, no differences for the [Ca^2+^]_i_ between the differentiated and undifferentiated POST-Ex cells and among the differentiated ones.

#### Superoxide production and general oxidation state

The myoblasts showed different level of superoxide production (Table 3). While the myoblasts from subject #1 maintained the same ${\text{O}}_{2}^{•-}$ levels, those from subjects #2, #3, and #4 showed significant decreases (*p* ≤ 0.0001); conversely, those from subjects #5 and #6 showed significant increases in ${\text{O}}_{2}^{•-}$ production (*p* ≤ 0.0001) after the exercise training. The increases in ${\text{O}}_{2}^{•-}$ production here were about 15% with respect to the control production, while the decreases ranged from 12 to 67% (Table 3).

**Table 3:** **Superoxide anion detection in the control (PRE-exercise) and after the low-moderate exercise conditioned (POST-exercise) satellite cells, as revealed by NBT dye fluorescence**.

**Subject**	**NBT dye fluorescence (mean** ±**SD)**		**Variation^a^(%)**
	**PRE-exercise**	**POST-exercise**	
#1	0.170 ± 0.030	0.180 ± 0.011	0
#2	0.132 ± 0.012	0.114 ± 0.006 §§	−14
#3	0.139 ± 0.004	0.045 ± 0.001 §§	−67
#4	0.080 ± 0.004	0.070 ± 0.002 §§	−12.5
#5	0.060 ± 0.002	0.070 ± 0.003 §§	+16
#6	0.106 ± 0.003	0.125 ± 0.004 §§	+18

a *percentage of variation with respect to PRE-Ex data (assumed as 100%), § significantly decreased or increased ${\text{O}}_{2}^{•-}$ radical level for POST-Ex vs. PRE-Ex (p < 0.001)*.

**Figure 2:**
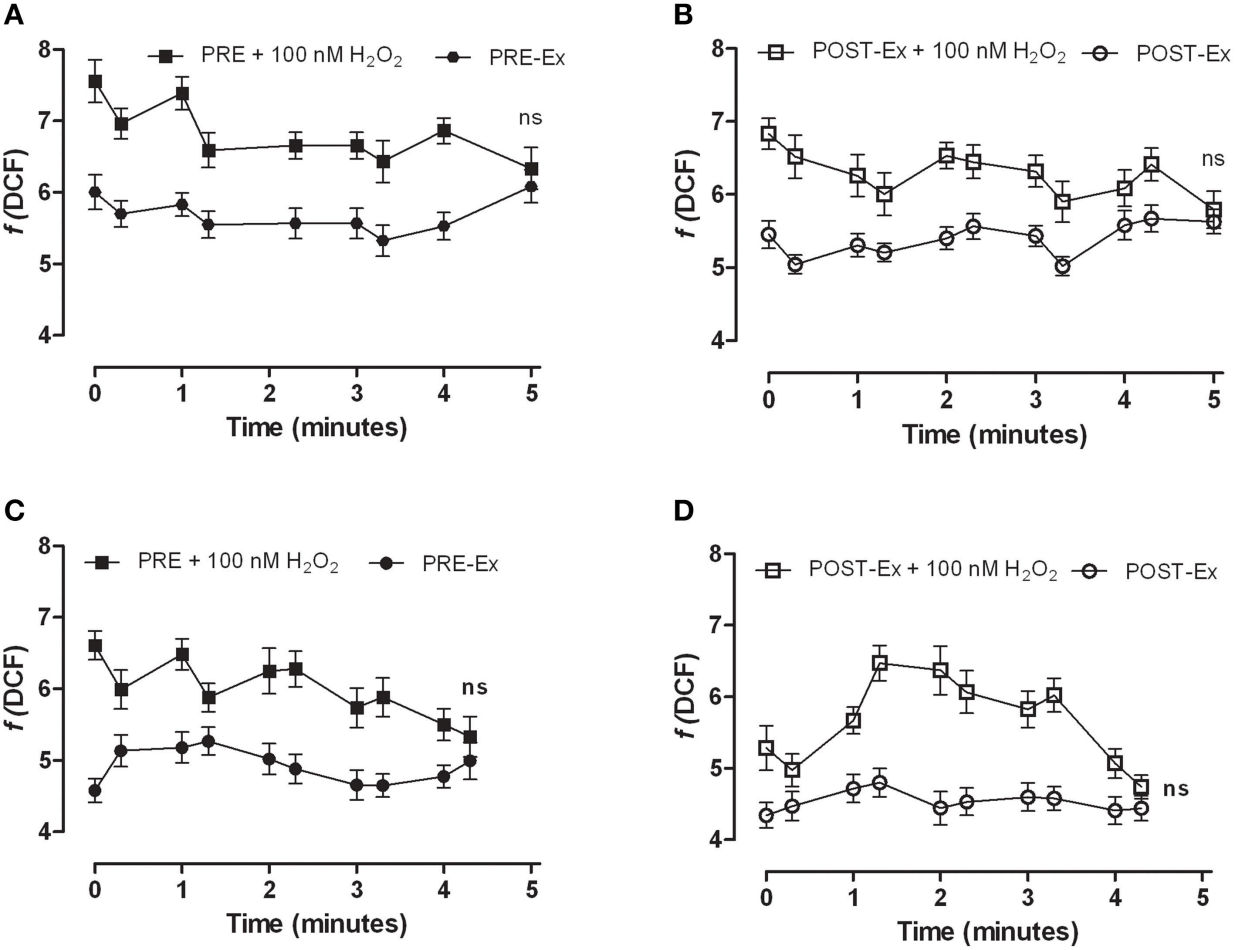
**Kinetics of DCF fluorescence in the control (PRE-Ex; A, C) and POST-Ex (B, D) myoblasts obtained from the skeletal muscle of the women that saw both decreased (A, B) and increased (C, D) ${\text{O}}_{2}^{•-}$ production, without and with addition of 100 nM H_2_O_2_ (as indicated)**. The H_2_O_2_ was added at 0 min in dedicated samples (+100 nM H_2_O_2_). Data came from three independent experiments. All of the ±H_2_O_2_ data points were significantly different (*p* ≤ 0.0001), except after 5 min ns, not significant.

The analysis of general oxidation state was conducted using DCF fluorescence. The addition of 100 nM H_2_O_2_ to the myoblasts loaded with DCF resulted in rapid increases in fluorescence that returned to basal level within 5 min, in the samples with both decreased and increased ${\text{O}}_{2}^{•-}$ production (Figure 2). Of note, the POST-Ex cell populations showing reduced superoxide anion production showed also a less amount of ROS at basal level, as revealed by 10% less DCF fluorescence with respect to that measured at PRE-Ex, even if not significant. In fact, the DCF fluorescence of POST-Ex vs. PRE-Ex at 0 min was 5.4 ± 0.7 vs. 6.0 ± 0.9 (Figure 2, confront Figure 2B vs. Figure 2A). The POST-Ex myoblasts showing increased superoxide anion production showed similar amounts of DCF fluorescent dye with respect to the PRE-Ex (4.6 ± 0.6 vs. 4.3 ± 0.7, not significant, Figures 2C,D).

#### Antioxidant enzyme activity

**Figure 3:**
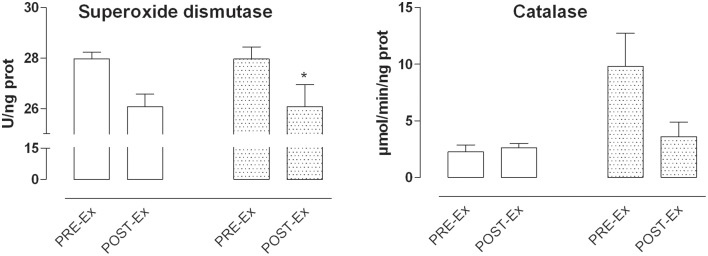
**Superoxide dismutase and Catalase activity**. In the Figure is shown a representative example of enzyme activities. The activity of Superoxide dismutase showed similar level on myoblasts with decreased ${\text{O}}_{2}^{•-}$ production (empty bars) while it was reduced in myoblasts showing increased ${\text{O}}_{2}^{•-}$ production (dotted bars) with respect to PRE-Ex (^*^p ≤ 0.05). The Catalase activity was similar among the PRE-Ex and POST-Ex cells, despite the ${\text{O}}_{2}^{•-}$ production.

The activity of antioxidant enzymes Superoxide dismutase and Catalase were determined on cytosolic fractions of PRE-Ex and POST-Ex undifferentiated cells both in population with reduced and increased superoxide anion production (Figure 3). The myoblasts with increased ${\text{O}}_{2}^{•-}$ production (empty bars) showed no variation of both enzyme activity while those with decreased ${\text{O}}_{2}^{•-}$ production (dotted bars) showed a significant reduction of Superoxide dismutase activity (*p* ≤ 0.05) and no significant change of Catalase activity.

#### Transmembrane mitochondrial potential

**Figure 4:**
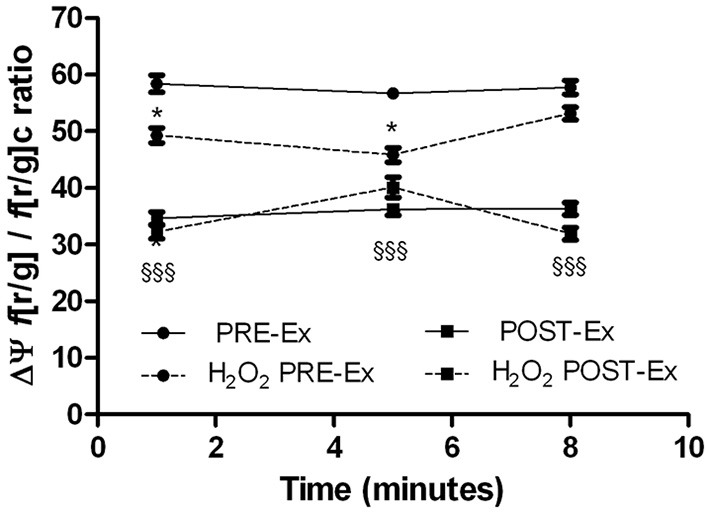
**Kinetics of the JC1 red/green fluorescence ratio variations as indirect measures of the transmembrane mitochondrial potential (ΔΨ) of myotubes obtained from skeletal muscle of female subjects for PRE-Ex and POST-Ex, without and with addition of 100 nM H_2_O_2_ (as indicated)**. Data came from three independent experiments. The treatment with H_2_O_2_ produced significant ΔΨ variation only in PRE-Ex cells (^*^p ≤ 0.05). The condition PRE-Ex vs. POST-Ex resulted significant only in untreated cells (§§§, *p* ≤ 0.0001).

The undifferentiated and differentiated PRE-Ex and POST-Ex cells showed stable transmembrane mitochondrial potentials (measured as the f[r/g]/f[r/g]_*c*_ ratio for JC-1; ΔΨ_mit_), which was reversibly depolarized (in a range of 10–20%) under the oxidative stimulus of 100 nM H_2_O_2_ (data not shown). Acute stimulation with the H_2_O_2_ induced less mitochondrial depolarization of the POST-Ex myotubes than was seen PRE-Ex, even if the depolarization levels were not significantly different (data not shown). The transmembrane mitochondrial potential of the myoblasts producing more superoxide anion provided an exception here: the PRE-Ex ΔΨ_mit_ showed H_2_O_2_-dependent depolarization, as previously described, while the POST-Ex ΔΨ_mit_ was stable with this addition of H_2_O_2_ (Figure 4).

### Epigenetic profile induced by exercise training

**Figure 5:**
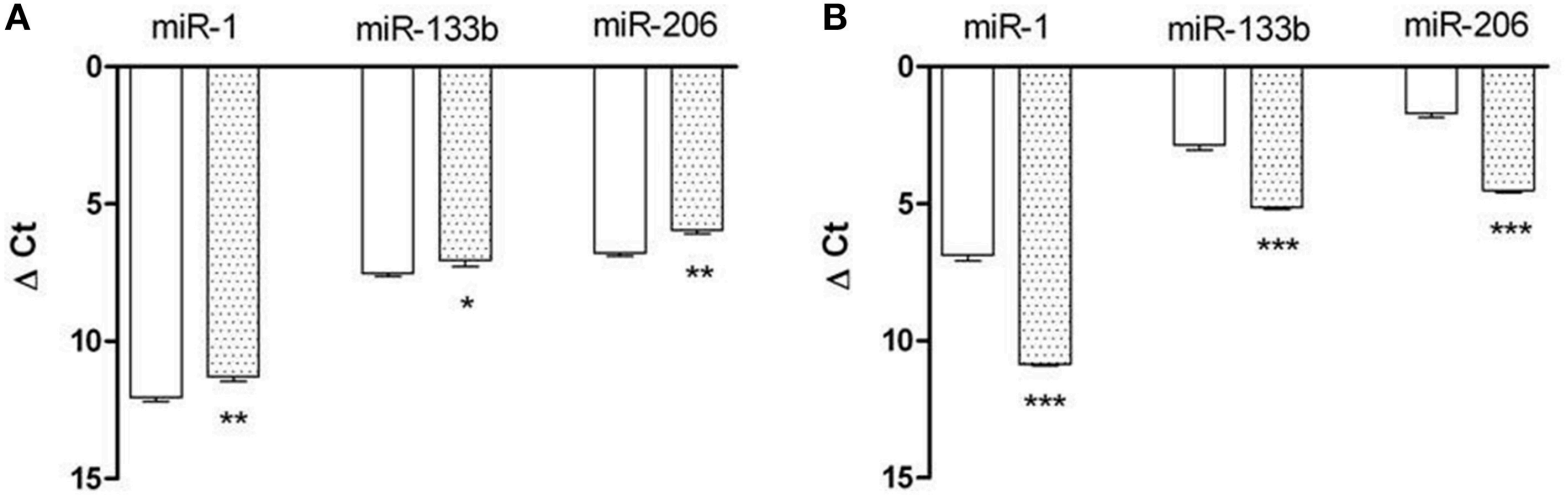
**Epigenetic signatures of miRNA expression**. Relative expression of miR-1, miR-133b, and miR-206 (as indicated) in undifferentiated cells obtained from skeletal muscle of female subjects that saw both decreased **(A)** and increased **(B)** ${\text{O}}_{2}^{•-}$ production before (empty bars) and after (dotted bars) exercise training. Data came from three independent experiments, each performed in triplicate. ^*^, *p* < 0.05; ^**^, *p* < 0.01; ^***^, *p* < 0.0001.

The analysis of the expression of miRNAs in the POST-Ex myoblasts showed an up-regulation of miR-1, miR133b, and miR206 respect to PRE-Ex in samples with decreased ${\text{O}}_{2}^{•-}$ production conversely we found a down-regulation of all miRNAs tested in samples with increased ${\text{O}}_{2}^{•-}$ production (Figure 5).

## Discussion

We have analyzed here skeletal muscle regeneration in adult women after low-to-moderate exercise training with specific attention paid to oxidative status. Recently, molecular studies in humans, highlighted that fusion of myogenic cells is triggered by endurance exercise-induced muscle plasticity (Frese et al., 2015)

At the cellular level, the fusion process is characterized by the alignment/fusion of myoblast membranes and cytoskeleton/cytoplasm rearrangements which results in the formation of nascent myotubes. Many studies of *in vitro* skeletal myogenesis have shown that myoblast fusion is regulated by calcium-increase in myoblasts before myotube formation (Constantin et al., 1996). We recorded an increased [Ca^2+^]_i_ in the POST-Ex myoblats that could be at the base of their increased ability to fuse to each other to form myotubes (Antigny et al., 2014). In fact, the fusion index significantly increased after the exercise training (POST-Ex) despite the ${\text{O}}_{2}^{•-}$ production, along with a trend to a reduction in the levels of desmin-positive cells that did not fuse in the differentiation media.

The data from the literature are consistent with the observation that intracellular ROS generation by contracting skeletal muscle increases by two-four–fold during contraction (Jackson et al., 2007). These ROS are derived through different biochemical pathways, and in particular by mitochondrial activity.

In fact, during aerobic training, the enzymatic activity of this electron transfer shifts from complex IV to complex III (maximal ADP-stimulated respiration), which improves the efficiency of the mitochondria for the production of ATP and the reduction of ${\text{O}}_{2}^{•-}$ (Di Meo and Venditti, 2001; Muller et al., 2004; Kozlov et al., 2005; Quinlan et al., 2013).

The results here for the satellite cell populations obtained after this low altitude exercise training suggested that the exercise linked to training provoked redox imbalance in some manner, mainly reducing ${\text{O}}_{2}^{•-}$ production. These data thus showed that in the satellite cell populations of three of the six subjects there was significantly reduced ${\text{O}}_{2}^{•-}$ production, for one of the six there was no change, and for two of the six there was about a 15% increase in the ${\text{O}}_{2}^{•-}$ production, as a relatively small amount. Albeit the myoblasts from two subjects increased cellular ${\text{O}}_{2}^{•-}$ production, this was linked to reduced superoxide dismutase activity. This reduction could be due to both the involvement of the enzyme in the oxidant reduction activity or in the partial inhibition of the dismutase enzyme.

The ROS species produced physiologically during exercise can stimulate important physiological mechanisms. For instance, there can be reversible oxidation of exposed protein thiols of the amino-acid cysteine in the ryanodine receptor, which governs correct excitation-contraction coupling (Fulle et al., 2007). Other examples include stimulation of mitochondrial biogenesis (Powers et al., 2011), up-regulation of antioxidant defenses (Gomez-Cabrera et al., 2008), expression of several genes for muscle hypertrophy (Powers et al., 2010), management of optimum muscle contractility (Reid et al., 1985), and muscle fatigue (Morillas-Ruiz et al., 2005).

Moreover, the data on general cellular peroxidation performed using DCF fluorescence, revealed that the presence of increased ${\text{O}}_{2}^{•-}$ production did not match with an establishment of oxidative stress. In fact, the POST-Ex cell populations showing increased ${\text{O}}_{2}^{•-}$ production, showed similar amount of DCF fluorescence with respect to PRE-Ex while those with reduced ${\text{O}}_{2}^{•-}$ production showed about 10% less amounts of the fluorescent dye DCF with respect to the PRE-Ex, albeit it not reached significant statistical differences. After the addition of H_2_O_2_ as external oxidant to mimic acute oxidative stress, all the cells completely reduced the ROS at the control levels in 5 min, as shown by the kinetics of DCF fluorescence.

The ${\text{O}}_{2}^{•-}$ radical is rapidly converted into the cell by the superoxide dismutases, to the more stable H_2_O_2_ (Abele et al., 2002). This H_2_O_2_ then undergoes specific degradation by catalase (Sullivan-Gunn and Lewandowski, 2013). The measurement of Catalase activity showed no significant differences among cell populations despite the level of ${\text{O}}_{2}^{•-}$ production, suggesting that probably the cells did not undergo the oxidative stress. The mitochondria are the main source of ${\text{O}}_{2}^{•-}$ production; in addition there are other intracellular sources, such as the sarcoplasmic reticulum-associated and plasma-membrane-associated NAD(P)H oxidases, whereby the latter release ${\text{O}}_{2}^{•-}$ mainly into the extracellular space, so they would be less important for intracellular ${\text{O}}_{2}^{•-}$ production. Although we cannot exactly distinguish the sources of increased ${\text{O}}_{2}^{•-}$ in our samples, we think that it could depend on the decreased superoxide dismutase activity and not on the impaired electron transfer shifts from mitochondrial complexes. In fact, the analysis of the mitochondrial transmembrane potential, ΔΨ_mit_, suggested that the cell populations with decreased superoxide production after the exercise training showed the same levels of ΔΨ_mit_ under the PRE-Ex and POST-Ex conditions. Acute stimulation with the H_2_O_2_ induced less mitochondrial depolarization of the POST-Ex myotubes than was seen PRE-Ex, which demonstrates potentially more efficient mitochondrial regulation. The investigation of ΔΨ_mit_ in the cell populations with increased superoxide production showed that albeit some POST-Ex myotubes were more depolarized than their PRE-Ex controls, the depolarizing insult with H_2_O_2_ did not provoke further variations. It could be that these mitochondrial potential were fixed as in a protective asset (Starkov, 1997). This might be linked to the effectiveness of the exercise training, which would adapt the myotubes to counteract oxidation-dependent depolarization and thus avoid its eventual negative consequences. In this manner, the mitochondrial functionality and the ATP production would remain optimal.

The miRNA analysis of myoblasts revealed a particular signature of this low-to-moderate training at low altitude in relation to the oxidant production. In fact, the increased accumulation of ${\text{O}}_{2}^{•-}$ in myoblasts occurred along with down-regulation of miR-1, miR-133b, and miR-206 expression while these miRNAs were up-regulated in samples with increased ${\text{O}}_{2}^{•-}$ production. miR-1 pushes cells toward apoptosis by inhibiting the heat shock proteins 60 and 70 which inhibit the mitochondrial apoptosis pathway, miR-133 acts in an opposite way through the repression of caspase nine. Interestingly, the coherent up- or down-regulation of miR-1 and miR-133b, as found in all our samples despite the ${\text{O}}_{2}^{•-}$ production, suggested that apoptosis was switched off (Xu et al., 2007). Moreover, we noted that when miRNA-1, miRNA-133b and miRNA-206 were up-regulated the cells showed decreased level of ${\text{O}}_{2}^{•-}$ production, on the contrary when down-regulated, increased level of ${\text{O}}_{2}^{•-}$ production. It could be possible that in female human myoblasts these miRNAs are specifically sensible to ${\text{O}}_{2}^{•-}$ presence. Moreover, the down-regulation of miRNA-1, miRNA-133b, and miRNA-206 has been correlated with skeletal muscle inflammatory (Georgantas et al., 2014). We think that in this muscle condition among oxidant species it could be present increased ${\text{O}}_{2}^{•-}$ production that could be responsible for these miRNA regulation and this scenario could be managed during female low training intensity session.

## Conclusions

The low to moderate intensity training has been able to stimulate the regeneration of female skeletal muscle. It induced mainly a decrease of ${\text{O}}_{2}^{•-}$ production, an increase of human myoblasts fusion index along with [Ca^2+^]_i_ increase. The ${\text{O}}_{2}^{•-}$ production could regulate the miRNA-1, miRNA 133b, and miRNA-206 expression without affecting the myoblast differentiation.

## Author contributions

TP designed the project, realised calcium imaging experiments, wrote the manuscript, analyzed and discussed the data. ED performed experiments on oxidative status and miRNA regulation. RM managed cell cultures, performed experiments on oxidative status, analyzed and discussed the data. CD trained the volunteers and discussed the data. AR performed experiments on oxidative status. GF discussed the data. SF analyzed and discussed the data.

### Conflict of interest statement

The authors declare that the research was conducted in the absence of any commercial or financial relationships that could be construed as a potential conflict of interest.

## Abbreviations

a.s.l.: altitude sea level
[Ca^2+^]_i_: intracellular calcium concentration
Cat: catalase
DCF-DA, 2^′^,7^′^: Dichlorodihydrofluorescein diacetate
JC-1, 5,5^′^,6,6^′^-Tetrachloro-1,1^′^, 3,3^′^: tetraethylbenzimidazolylcarbocyanine
H_2_O_2_: hydrogen peroxide; iodide/chloride
MHC: Myosin Heavy Chain
miRNA: micro ribonucleic acid
${\text{O}}_{2}^{•-}$: superoxide anion
PBS: Dulbecco's phosphate-buffered saline
ROS: Reactive oxygen species
SOD: superoxide dismutase.
